# A case of rectal metastasis of prostate cancer mimicking extramural growth-type rectal tumor

**DOI:** 10.1093/jscr/rjae314

**Published:** 2024-05-18

**Authors:** Shutaro Hike, Satoshi Endo, Masayuki Ota, Gaku Ohira, Michihiro Maruyama, Shunsuke Imanishi, Tetsuro Maruyama, Toru Tochigi, Hiroyuki Amagai, Tadashi Shiraishi, Atsushi Hirata, Hisashi Mamiya, Moe Iwata, Ayano Kakimoto, Hisahiro Matsubara

**Affiliations:** Department of Frontier Surgery, Graduate School of Medicine, Chiba University, Chiba 260-8670, Japan; Department of Frontier Surgery, Graduate School of Medicine, Chiba University, Chiba 260-8670, Japan; Department of Diagnostic Pathology, Graduate School of Medicine, Chiba University, Chiba 260-8670, Japan; Department of Frontier Surgery, Graduate School of Medicine, Chiba University, Chiba 260-8670, Japan; Department of Frontier Surgery, Graduate School of Medicine, Chiba University, Chiba 260-8670, Japan; Department of Frontier Surgery, Graduate School of Medicine, Chiba University, Chiba 260-8670, Japan; Department of Frontier Surgery, Graduate School of Medicine, Chiba University, Chiba 260-8670, Japan; Department of Frontier Surgery, Graduate School of Medicine, Chiba University, Chiba 260-8670, Japan; Department of Frontier Surgery, Graduate School of Medicine, Chiba University, Chiba 260-8670, Japan; Department of Frontier Surgery, Graduate School of Medicine, Chiba University, Chiba 260-8670, Japan; Department of Frontier Surgery, Graduate School of Medicine, Chiba University, Chiba 260-8670, Japan; Department of Frontier Surgery, Graduate School of Medicine, Chiba University, Chiba 260-8670, Japan; Department of Frontier Surgery, Graduate School of Medicine, Chiba University, Chiba 260-8670, Japan; Department of Frontier Surgery, Graduate School of Medicine, Chiba University, Chiba 260-8670, Japan; Department of Frontier Surgery, Graduate School of Medicine, Chiba University, Chiba 260-8670, Japan

**Keywords:** prostate cancer, rectal metastasis, extramural growth-type rectal tumor, resection

## Abstract

Rectal metastases of prostate cancer are rare and may be difficult to diagnose. In this report, we describe a case in which an extramural growth-type rectal tumor was resected and pathologically diagnosed as prostate cancer metastasis. A 70-year-old man on hormone therapy for prostate cancer with seminal vesicle invasion and pelvic lymph node metastasis was referred to our department after an imaging scan showed an extramural growth-type rectal tumor. Endoscopic ultrasound-guided fine needle aspiration was considered for diagnosis, but the patient preferred an early resection without the exam, so surgery was performed. Histopathological examination revealed that the lesion was in the adventitia of the rectum and metastasis of prostate cancer. Metastatic lesions of prostate cancer are not indicated for resection. A detailed preoperative study with the possibility of prostate cancer metastasis in mind is necessary because it is relevant to choosing the treatment strategy.

## Introduction

The commonest sites of prostate cancer metastasis are the pelvic lymph nodes, bones, liver, and lungs. Rectal metastasis is relatively rare and may be difficult to diagnose [1]. Unlike localized rectal tumors, metastatic lesions of prostate cancer are not indicated for resection and require a detailed preoperative review because the treatment strategy is different. We herein report a relatively rare case of rectal metastasis of prostate cancer that mimics an extramural growth-type rectal tumor.

## Case report

**Figure 1:**
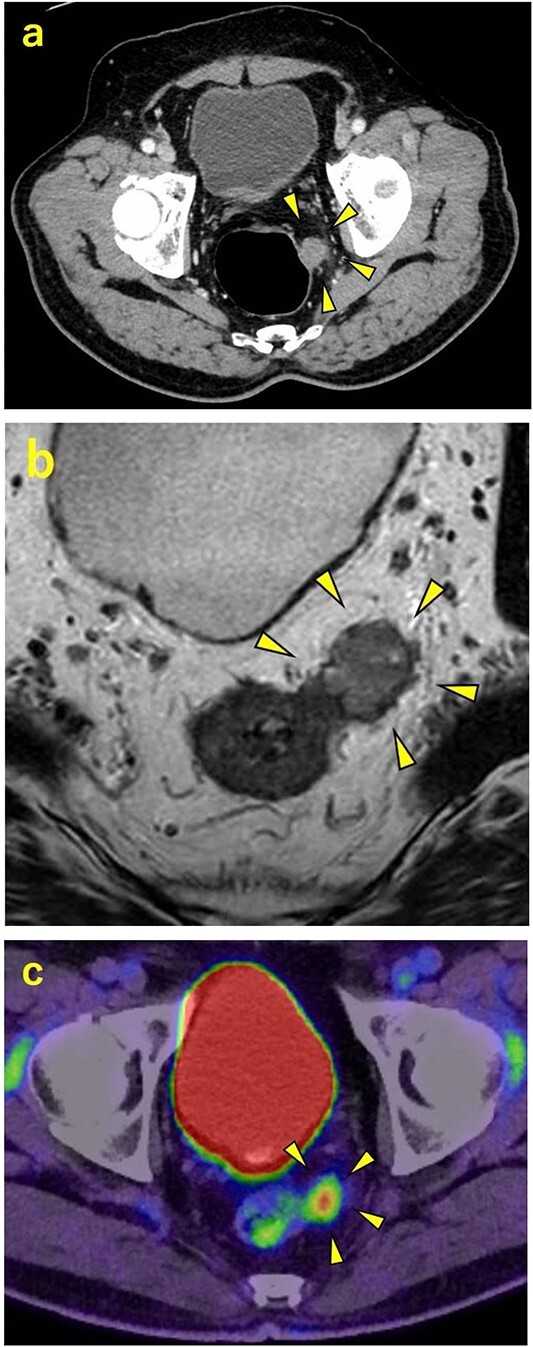
Contrast-enhanced CT, contrast-enhanced MRI, and PET. (a) CT showing a 20-mm large extramural growth-type mass on the left wall of the rectum, 9 cm from the anal verge. (b) MRI showing that the mass may have originated from the muscularis propria of the rectum. (c) PET showing an accumulation in the mass with a maximum standardized uptake value of 5.96.

**Figure 2:**
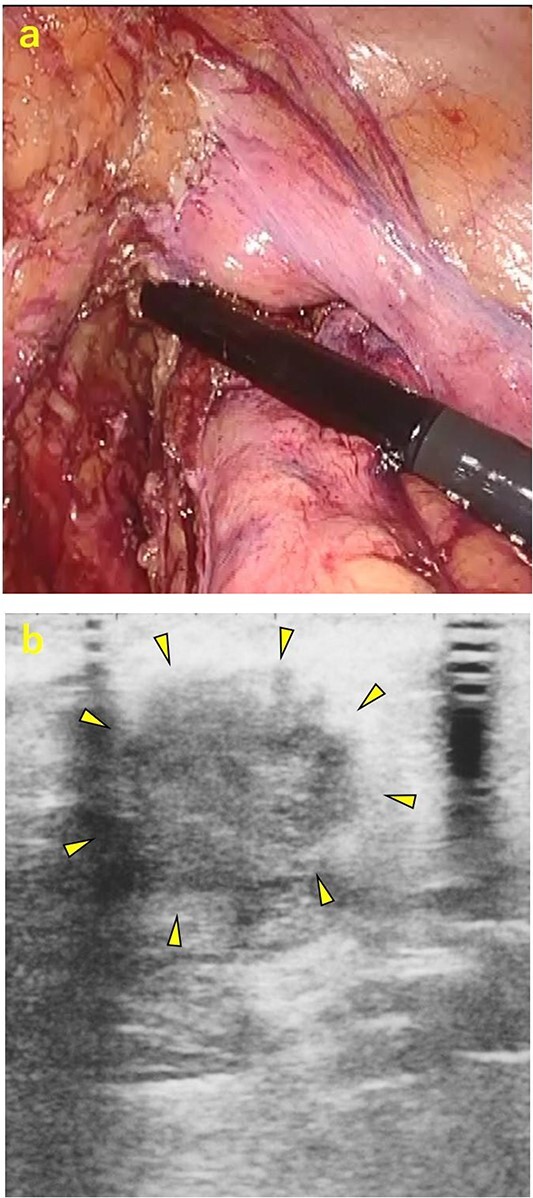
Intraoperative ultrasonography. (a) A laparoscopic ultrasonography probe was applied to the mesorectum to identify the mass. (b) A hypoechoic area of ~20 mm

**Figure 3:**
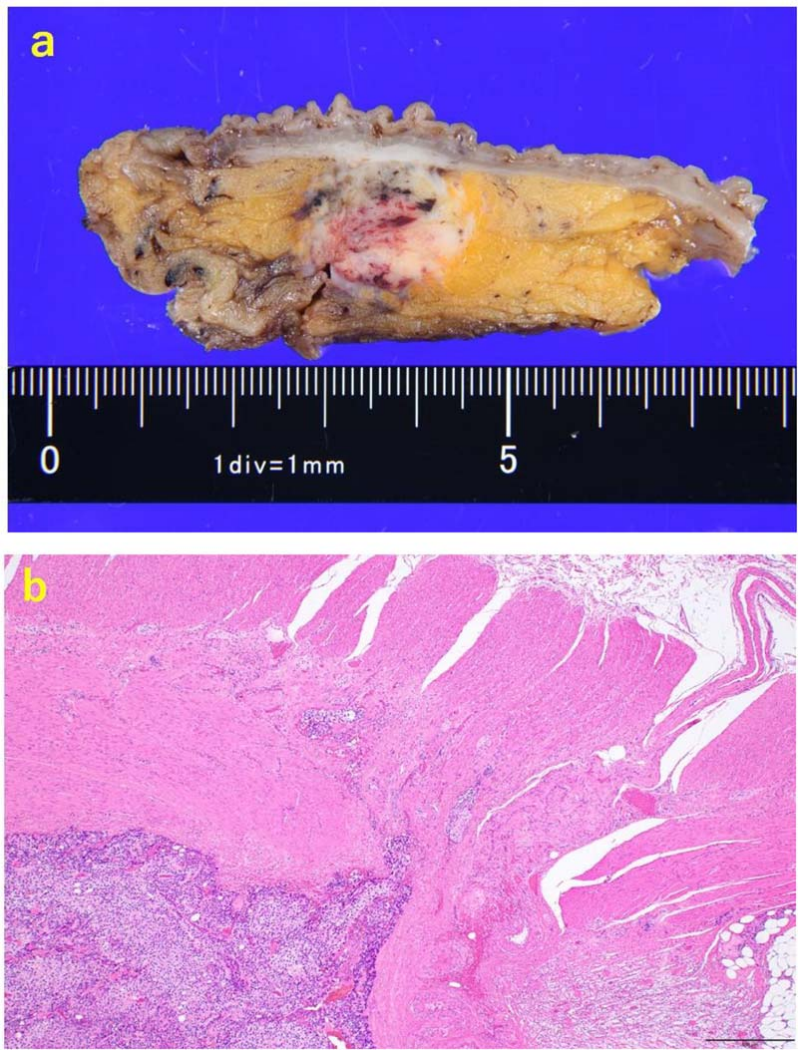
The resected specimen. (a) The resected specimen: a well-defined 18 × 16 mm large borderline mass predominantly located on the lower rectal adventitia. (b) HE (×40) specimen showing mass compressing the muscularis propria after an increase in size.

**Figure 4:**
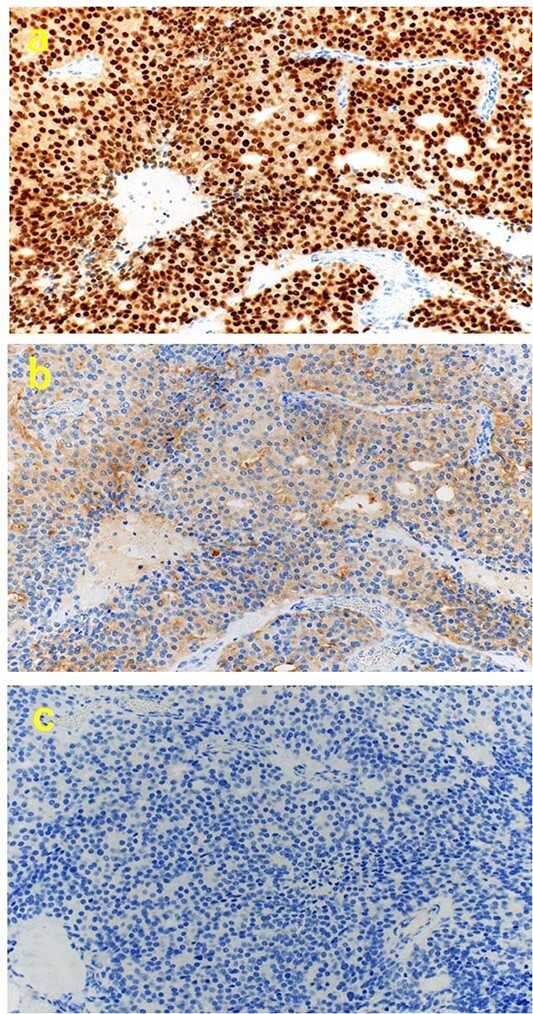
Immunohistochemical findings. (a) (×200) positive for NKX3.1, (b) (×200) partially positive for PSA, and (c) (×200) negative for CDX2.

A 70-year-old man was referred to our urology department for prostate cancer based on a mass contiguous with the rectum seen on an imaging scan. He had a Gleason score of 4 + 5, seminal vesicle invasion, and pelvic lymph node metastasis. A month of combined androgen blockade therapy had reduced his prostate-specific antigen (PSA) levels from 25.29 to 10.81 ng/ml. He was diagnosed with rectal cancer at 68 years old and had undergone an endoscopic mucosal resection (EMR) and curative resection. The tumor marker levels were as follows: carcinoembryonic antigen, 2.8 ng/ml and carbohydrate antigen 19–9, 25.4 U/ml. Lower gastrointestinal endoscopy revealed only an EMR scar in the rectum. Computed tomography (CT) revealed a 20-mm large extramural growth-type mass on the left wall of the rectum, 9 cm from the anal verge (Fig. 1a). The mass had a weak enhancing effect and somewhat irregular limbus, and was continuous with the rectus muscularis. The mass was close to the ureter-hypogastric fascia and was suspected to have invaded the pelvic plexus. We observed an enhancing effect in the left lobe of the prostate, and an enlarged left lateral lymph node. No distant metastases were found in other organs, including the bones. Magnetic resonance imaging (MRI) revealed that the mass may have originated from the muscularis propria of the rectum (Fig. 1b). The prostate carcinoma partially touched the rectum beyond the capsule, with no evidence of invasion. 18F-fluorodeoxyglucose-positron emission tomography-CT (FDG PET-CT) showed accumulation in the mass, with a maximum standardized uptake value of 5.96. There was no accumulation in any other organ, including the prostate and pelvic lymph nodes (Fig. 1c). After examination, gastrointestinal stromal tumor (GIST), neuroendocrine tumor (NET), and lymph node metastatic recurrence of rectal cancer were considered as differential diagnoses. Endoscopic ultrasound-guided fine needle aspiration (EUS-FNA) was scheduled for pathological diagnosis. However, since the patient denied the EUS-FNA and preferred an early resection, we performed only surgery. During the laparoscopic low anterior resection, a laparoscopic ultrasonography probe was applied to the mesorectum to identify the mass (Fig. 2a). A hypoechoic area was observed in the left mesorectum (Fig. 2b). Since we suspected invasion into the ureter-hypogastric fascia, the area around the lesion was resected, with partial merging of the pelvic plexus. Postoperatively, the patient had a mildly relieved neurogenic bladder and was discharged on postoperative Day 8. The resected specimen showed a well-defined 18 × 16 mm large borderline mass predominantly on the lower rectal adventitia (Fig. 3a). The lesion was non-contiguous and located contralateral to the EMR scar. Histopathological findings revealed that the mass was poorly differentiated carcinoma and grew to compress the muscularis propria (Fig. 3b). The tumor was immunohistochemically positive for CK AE1/AE3 and NKX3.1 (Fig. 4a); partially positive for PSA (Fig. 4b); and negative for CK7, CK20, CDX2 (Fig. 4c), TTF1, chromogranin A, synaptophysin, CD56, C-KIT, and SOX10. Therefore, the tumor was diagnosed with rectal metastasis of prostate cancer. Cancer metastases of similar histology were also found in other resected lymph nodes. Enzalutamide was started 2 months postoperatively, but due to tumor and metastatic lymph node enlargement, docetaxel treatment was started at 5 months postoperatively. However, the disease continued to progress, and he died 10 months after surgery.

## Discussion

We herein report a relatively rare case of rectal metastasis of prostate cancer mimicking an extramural growth-type rectal tumor. We recognized its localization, resected it laparoscopically with intraoperative echography, and diagnosed rectal metastasis of prostate cancer on pathology. Here, preoperative CT and MRI findings suggested that the solitary extramural growth lesion arose from the intrinsic muscularis propria of the rectum, and FDG PET-CT showed accumulation only in that lesion and no other organ, including the prostate gland. Although this was a case of prostate cancer with seminal vesicle invasion and pelvic lymph node metastasis, we thought that GIST, NET, or lymph node metastatic recurrence of rectal cancer was more likely than solitary rectal metastasis of prostate cancer. The most likely mode of rectal metastasis in this case was lymphatic, as microscopic metastasis of prostate cancer was also observed in the resected mesorectal lymph node. Studies show that prostate cancer with seminal vesicle invasion has a high rate of metastasis to mesorectal lymph nodes [2], and we hypothesized that prostate cancer metastasized to the mesorectal lymph nodes near the rectum, invaded the surrounding area, and became close to the muscularis propria of the rectum, mimicking an extramural growing rectal tumor. Data on the utility of FDG PET-CT in prostate cancer are lacking, but the sensitivity in untreated primary prostate cancer is 33% [3], and the FDG accumulation rate is generally low [4–6]. Furthermore, FDG accumulation in metastatic lesions tends to decrease with androgen deprivation therapy, but there are variations in individual lesions [7]. However, there are no reports of accumulation in only one metastatic lesion, as in the present case, which is unusual and misleading. In our case, the resection was performed without preoperative definitive diagnosis by EUS-FNA at the patient’s request, but surgery could have been avoided if the diagnosis of metastasis from prostate cancer was made using biopsy. In pathological diagnosis, NKX3.1 immunostaining is a useful marker for prostate origin in addition to PSA, with a reported sensitivity of 98.6% and specificity of 99.7% in metastatic lesions [8]. Moreover, a highly accurate diagnosis can be expected if tissue samples are available. In case of a rectal tumor with a history of prostate cancer and the possibility of metastasis, it is necessary to perform a detailed preoperative study, including biopsy findings.
